# COVID-19-Related Immune Activation and Capsular Contracture: A Cohort Study of Infection, Vaccination, and Explantation

**DOI:** 10.1007/s00266-026-05965-2

**Published:** 2026-07-01

**Authors:** Gloria S Lazzaretti, Denis S Valente, Lucas P Steffen, Vinicius Kayser, Rafaela K Zanella

**Affiliations:** 1 Graduate Program in Health Sciences, Federal University of Medical Sciences of Porto Alegre, Porto Alegre, Brazil; 2 Plastic Surgery, Federal University of Medical Sciences of Porto Alegre, Rua Antonio Carlos Berta 475- 7th Floor, Porto Alegre, RS 91340-020 Brazil; 3 School of Medicine, Pontifical University of Rio Grande do Sul, Porto Alegre, Brazil

**Keywords:** Capsular contracture, Breast augmentation, COVID-19, Immune activation, Explantation, Cohort study

## Abstract

**Background:**

Capsular contracture, a prevalent complication following breast augmentation, is characterized by pain, implant distortion, and often necessitates explantation. While its multifactorial pathogenesis involves bacterial biofilms and implant characteristics, the influence of immune activation, particularly in the context of COVID-19 infection and vaccination, remains underexplored. Growing evidence suggests COVID-19 vaccination can trigger immune-mediated reactions, including delayed inflammatory responses to dermal fillers. Given that capsular contracture is a fibro-inflammatory response, this study investigated whether COVID-19-related immune activation influenced the time from breast implantation to explantation due to severe capsular contracture.

**Methods:**

This retrospective cohort study analyzed patients from a single private practice who underwent breast implant removal due to Baker grade III or IV capsular contracture. Two historical cohorts were compared: Cohort 1 (pre-pandemic: January 2016 – December 2019) and Cohort 2 (post-pandemic: January 2021 – December 2024).The primary outcome, time from implantation to explantation, was analyzed using Kaplan-Meier survival curves, log-rank tests, and a multivariate Cox proportional hazards model, adjusting for confounders. Ethical approval was obtained (CAAE: 35154720.2.0000.5330).

**Results:**

A total of 115 patients were included (47 in Cohort 1, 68 in Cohort 2). Baseline characteristics were comparable between groups. The time from breast implantation to explantation due to Baker grade III/IV capsular contracture was significantly shorter in the post-pandemic cohort (4.9 ± 1.3 years) compared to the pre-pandemic cohort (8.2 ± 1.5 years, *p *< 0.001). The Kaplan-Meier estimated median time to explantation was 5.0 years (95% CI: 4.6–5.3) for the post-pandemic cohort versus 8.0 years (95% CI: 7.6–8.4) for the pre-pandemic cohort (*p *< 0.001). Multivariate Cox analysis showed a hazard ratio of 2.3 (95% CI: 1.5-3.5, *p *< 0.001) for earlier explantation in the post-pandemic cohort, independent of confounders. No significant differences were found in the proportion of Baker IV contractures or primary complaints.

**Conclusion:**

While this study does not establish causation, the observed reduction in the time to explantation in the post-pandemic cohort compared to the pre-pandemic cohort suggests that external factors, including potential immunological responses to the COVID-19 vaccine or infection, may have influenced the progression of capsular contracture. This information is relevant for clinical practice and has implications for patient management, informed consent, and medicolegal assessment of implant-related complications.

**Level of Evidence III:**

This journal requires that authors assign a level of evidence to each article. For a full description of these Evidence-Based Medicine ratings, please refer to the Table of Contents or the online Instructions to Authors www.springer.com/00266.

## Introduction

Capsular contracture is one of the most frequent complications following breast augmentation, often leading to pain, implant distortion, and the need for surgical explantation [1]. While its pathogenesis is multifactorial, involving bacterial biofilms, implant surface characteristics, and host immune response, the factors influencing its progression are not completely understood [2]. Given the widespread immune activation triggered by COVID-19 vaccination and infection, it is important to investigate whether these factors may have influenced the incidence or severity of these conditions [3]. To date, only one study has explored the relationship between capsular contracture and COVID-19, specifically in prosthetic breast reconstruction [4].

The long-term impact of the COVID-19 pandemic on various medical conditions remains an area of active investigation; there is growing evidence that COVID-19 vaccination can trigger immune-mediated reactions in aesthetics, particularly in patients with dermal fillers [5]. Multiple reports have documented delayed inflammatory responses, including facial edema and nodular reactions, following vaccination [6]. These findings suggest that heightened immune surveillance may contribute to unexpected inflammatory processes in the presence of foreign materials. Since capsular contracture is a fibro-inflammatory response to breast implants, it is reasonable to question whether similar immune mechanisms could influence the progression of periprosthetic fibrosis. This external, patient-mediated factor may alter implant longevity and precipitate earlier reoperation. Our study aims to investigate whether the time from breast implantation to explantation due to severe capsular contracture has changed in the post-pandemic period. By analyzing two historical cohorts, one treated before COVID-19 and another after the global vaccination campaign, our research seeks to determine if a significant shift has occurred.

## Materials and Methods

This study analyzed patients from the senior author’s private practice who underwent breast implant removal due to Baker grade III or IV capsular contracture. This research included patients whose implants were placed either by the senior author or by other surgeons. Two historical cohorts were compared: Cohort 1 consisted of patients operated on between January 2016 and December 2019, and Cohort 2 included patients treated between January 2021 and December 2024. The primary objective was to assess differences in the time between breast implantation and surgical explantation and to explore potential associations with COVID-19 vaccination status.

This study was approved by the Institutional Ethics Committee and is registered with the Brazilian Ministry of Health under the number: CAAE:35154720.2.0000.5330. All patients provided written informed consent for the use of their medical records for research purposes.

### Patient Selection

Patients were identified from the senior author’s clinical records, with inclusion criteria being: (1) prior breast augmentation with silicone implants, (2) explantation due to Baker III or IV capsular contracture, and (3) availability of complete medical records documenting the date of implant placement and explantation, when available, retrospective histopathological reports were reviewed to characterize the capsule, specifically focusing on the presence of synovial metaplasia, thickness, and inflammatory infiltrate. Exclusion criteria included: (1) implant rupture diagnosed either prior to or during the surgery, (2) active infection or history of periprosthetic infection, (3) history of radiation therapy to the chest, (4) prior history of breast cancer or oncologic breast surgery, and (5) insufficient or incomplete medical documentation.

### Data Collection

Demographic and clinical data were collected, including patient age, BMI, smoking status, implant type, implant placement plane (subglandular or submuscular), and implant volume. The primary outcome was the duration between breast implantation and explantation. For patients in Cohort 2, COVID-19 vaccination status, including the number of doses received and the time since the last dose, was recorded. Furthermore, the specific vaccine platform (mRNA, adenoviral vector, or inactivated virus) was documented for each patient to account for varying degrees of systemic immune activation.

### Statistical Analysis

Baseline characteristics between the two cohorts were compared using independent t-tests for continuous variables (age, BMI) and Chi-square tests for categorical variables (smoking status, implant type, placement plane). The primary outcome, time from breast implantation to explantation, was analyzed using Kaplan–Meier survival curves and log-rank tests. A multivariate Cox proportional hazards model was used to adjust for potential confounders, including age, smoking status, implant type, and implant placement plane. A* p*-value < 0.05 was considered statistically significant.

## Results

A total of 115 patients were included in our study, with 47 in Cohort 1 (2016–2019) and 68 in Cohort 2 (2021–2024). Table 1 shows that the baseline characteristics of both groups were comparable, with no significant differences in age (35.8 ± 6.4 vs. 36.1 ± 5.9 years, *p* = 0.78), BMI (23.7 ± 2.8 vs. 24.1 ± 3.1 kg/m^2^, *p* = 0.54), or smoking status (25.5% vs. 26.5%, *p *= 0.89). All implants were silicone-filled with a texturized surface, and the mean implant volume was similar between cohorts (315 ± 50 vs. 320 ± 55 mL, *p* = 0.63). No differences were found in implant placement, with similar proportions of subglandular (29.8% vs. 27.9%, *p* = 0.81) and submuscular placement (70.2% vs. 72.1%, *p* = 0.81). Additional variables such as time since last childbirth and family history of autoimmune diseases were also comparable between groups. In Cohort 2, the mean number of COVID-19 vaccine doses was 2.8 ± 0.5, with a mean time since the last dose of 10.3 ± 3.4 months. Regarding vaccine platforms in Cohort 2, 47.4% received mRNA-based vaccines (BNT162b2), 30.5% received adenoviral vector vaccines (ChAdOx1 nCoV-19), and 22.1% received inactivated virus vaccines (CoronaVac). No significant difference in time to explantation was observed between vaccine platforms in a preliminary subgroup analysis.

**Table 1:** Baseline characteristics of patients in both cohorts

Characteristic	Cohort 1 (2016–2019) (*n* = 47)	Cohort 2 (2021–2024) (*n* = 68)	*p*-value
Age (years, mean ± SD)	35.8 ± 6.4	36.1 ± 5.9	0.78
BMI (kg/m2, mean ± SD)	23.7 ± 2.8	24.1 ± 3.1	0.54
Smokers (%)	12 (25.5%)	18 (26.5%)	0.89
Previous breast surgery (%)	9 (19.1%)	15 (22.1%)	0.71
Implant type	Silicone, texturized	Silicone, texturized	–
Implant volume (mL, mean ± SD)	315 ± 50	320 ± 55	0.63
Subglandular placement (%)	14 (29.8%)	19 (27.9%)	0.81
Submuscular placement (%)	33 (70.2%)	49 (72.1%)	0.01
Time since last childbirth (years, mean ± SD)	6.4 ± 3.2	6.2 ± 3.5	0.79
Family history of autoimmune disease (%)	7 (14.9%)	12 (17.6%)	0.72
Number of COVID-19 vaccine doses (mean + SD)	N/A	2.8 ± 0.5	–
Time since last vaccine dose (months, mean ± SD)	N/A	10.3 ± 3.4	–

Table 2 shows that the primary outcome, time from breast implantation to explantation due to Baker grade III/IV capsular contracture, was significantly shorter in the post-pandemic cohort compared to the pre-pandemic cohort (4.9 ± 1.3 vs. 8.2 ± 1.5 years, *p* < 0.001). The Kaplan–Meier estimated median time to explantation was also significantly lower in the post-pandemic cohort (5.0 years; 95% CI: 4.6–5.3) than in the pre-pandemic cohort (8.0 years; 95% CI: 7.6–8.4) (*p *< 0.001). Multivariate Cox proportional hazards analysis demonstrated a hazard ratio of 2.3 (95% CI: 1.5–3.5, *p *< 0.001) for earlier explantation in the post-pandemic cohort, even after adjusting for potential confounders. No significant differences were observed in the proportion of Baker IV contractures between cohorts (65.9% vs. 72.1%, *p *= 0.49) or in primary complaints leading to surgery, such as pain (53.2% vs. 64.7%, *p *= 0.22) or aesthetic concerns (31.9% vs. 26.5%, *p *= 0.52).

**Table 2:** Comparison of time from breast implantation to explantation and statistical analysis

Outcome	Cohort 1 (2016–2019) (*n* = 47)	Cohort 2 (2021–2024) (*n* = 68)	*p*-value
Years from implantation to explantation (mean ± SD)	8.2 ± 1.5	4.9 ± 1.3	< 0.001
Baker IV contracture (%)	31 (65.9%)	49 (72.1%)	0.49
Pain as primary complaint (%)	25 (53.2%)	44 (64.7%)	0.22
Aesthetic concern as primary complaint (%)	15 (31.9%)	18 (26.5%)	0.52
Localized swelling (%)	6 (12.8%)	13 (19.1%)	0.39
Kaplan-Meier estimated median time to explantation (years)	8.0 (95% CI: 7.6–8.4)	5.0 (95% CI: 4.6–5.3)	< 0.001
Hazard ratio for early explantation (Cox model)	–	2.3 (95% CI: 1.5–3.5)	< 0.001
Patients with prior COVID-19 infection (%)	N/A	28 (41.2%)	–
Patients reporting systemic symptoms post-vaccination (%)	N/A	15 (22.1%)	–

In the post-pandemic cohort, 41.2% of patients reported a prior COVID-19 infection, and 22.1% reported experiencing systemic symptoms following vaccination.

## Discussion

Capsular contracture remains a significant complication following breast augmentation, with multifactorial etiologies involving both mechanical and immunological factors [7]. Our study identified a substantial reduction in the time from breast implantation to explantation due to Baker grade III or IV contracture in the post-pandemic cohort compared to the pre-pandemic cohort. While previous research has primarily focused on bacterial biofilm formation and implant surface characteristics as key determinants of contracture development [8], our findings suggest the possibility of additional immunological influences accelerating this process in recent years. The observed decrease in contracture latency raises important questions regarding external factors that may have altered the natural course of this pathology, warranting further investigation.

The observed association between COVID-19-related immune activation and accelerated peri-implant fibrosis suggests a potential external, patient-mediated factor that may influence implant longevity; patients should note that such systemic events lie outside the direct control of the surgeon. Documentation that standard surgical technique and accepted perioperative protocols were followed, together with routine postoperative follow-up, provides an appropriate clinical response; additional onerous measures beyond established standards are not required solely on the basis of the observed association.

The significant difference in time to explantation between cohorts remained robust even after adjusting for confounding variables such as patient age, BMI, smoking status, implant placement, and implant volume. The immunological landscape following the COVID-19 pandemic has been profoundly altered by widespread vaccination programs and prior viral infections, which have been implicated in heightened inflammatory responses [9]. Studies on immune system activation following mRNA vaccination have demonstrated transient increases in pro-inflammatory cytokines, including IL-6 and TNF-*α*, which are known to play a role in fibrotic disorders [10]. Additionally, recent evidence suggests that SARS-CoV-2 infection itself may contribute to dysregulated immune responses, further complicating the understanding of post-pandemic fibrosis-related conditions [11].

Despite the lack of direct histopathological evidence linking COVID-19 vaccination or infection to accelerated capsular contracture, our findings of an increased prevalence of earlier-onset contracture in the post-pandemic Cohort suggests a potential association that cannot be overlooked. A growing body of literature has described cases of COVID-19 vaccine-related delayed inflammatory reactions in patients with dermal fillers, reinforcing the hypothesis that immune-mediated mechanisms could contribute to accelerated fibrosis [12, 13]. However, it remains unclear whether the reduction in contracture latency observed in our study is attributable to a specific immunological mechanism, changes in patient behavior (such as increased vigilance regarding implant-related complications), or other unidentified environmental factors [14].

Our findings reveal no statistically significant difference between the two cohorts in the proportion of Baker IV contractures or the primary patient-reported complaints prompting explantation. This suggests that, while the temporal dynamics of contracture development appear altered, the severity of clinical presentation remains consistent. Our observation may imply that, although earlier inflammatory events could accelerate the onset of clinically significant contracture, the final fibrotic burden at explantation is unchanged. We propose a threshold model to explain these findings, positing that inflammatory insults accelerate the timing of contracture onset without necessarily influencing the degree of ultimate fibrosis. These hypotheses underscore the necessity for mechanistic investigations incorporating histological, genetic, and immunological profiling of capsular tissue harvested from patients within both cohorts. Our findings necessitate a deeper immunophenotypic evaluation of these ‘accelerated’ capsules. While Baker Grade IV presentation remained consistent across cohorts, we hypothesize that post-pandemic capsules may exhibit a higher density of CD68+ macrophages and increased expression of TGF-*β*, reflecting a state of ‘primed’ fibrosis triggered by systemic immune surveillance.

It is important to acknowledge that our study is retrospective and has some limitations as selection biases and unmeasured confounders. Additionally, while vaccination data were collected, our study was not designed to establish a causal relationship between COVID-19 vaccination and capsular contracture onset. The lack of a control group of non-vaccinated individuals further limits the ability to draw strong inferences regarding vaccine-specific effects. Critically, the retrospective nature of this study precluded a standardized, prospective immunohistochemical comparison of explanted capsules between cohorts. Therefore, we cannot definitively state whether the accelerated fibrosis in Cohort 2 is qualitatively different (e.g., higher myofibroblast density) or simply a temporally compressed version of the standard fibro-inflammatory response. Furthermore, patient-reported histories of prior COVID-19 infection and systemic vaccine-related symptoms introduce recall bias, which may influence data accuracy.

The long-term impact of the COVID-19 pandemic on various medical conditions remains an area of active investigation [15]. Future research should incorporate prospective cohort designs with detailed immunohistochemical analyses of explanted capsules to investigate potential differences in inflammatory cell infiltration, cytokine profiles, and fibrotic gene expression. Additionally, studies evaluating the role of booster vaccine doses and potential dose-dependent effects on implant-related inflammation could be useful. As the landscape of implant-based breast surgery continues to evolve, understanding the immunological determinants of capsular contracture will help optimize patient outcomes and develop targeted therapeutic strategies.

## Conclusion

While this study does not establish causation, the observed reduction in the time to explantation in the post-pandemic cohort compared to the pre-pandemic cohort suggests that external factors, including potential immunological responses to the COVID-19 vaccine or infection, may have influenced the progression of capsular contracture. This information is relevant for clinical practice and has implications for patient management, informed consent, and medicolegal assessment of implant-related complications.

## Declarations

### Artificial Intelligence Statement

During the preparation of this work, the authors used the Gemini language model to facilitate the translation of the article from Portuguese to English and Paperpile for the management and formatting of references. The use of these tools was limited to enhancing language and streamlining citation; the authors reviewed and edited the content as needed and take full responsibility for the content of the publication.
